# Current practice and usual care of major cervical disorders in Korea: A cross-sectional study of Korean health insurance: Erratum

**DOI:** 10.1097/MD.0000000000016335

**Published:** 2019-06-28

In the article, “Current practice and usual care of major cervical disorders in Korea: A cross-sectional study of Korean health insurance”,^[1]^ which appears in issue 96, Issue 46 of *Medicine*, the rows in Table 3 were mislabeled. The correct table is below.

**Table 3:**
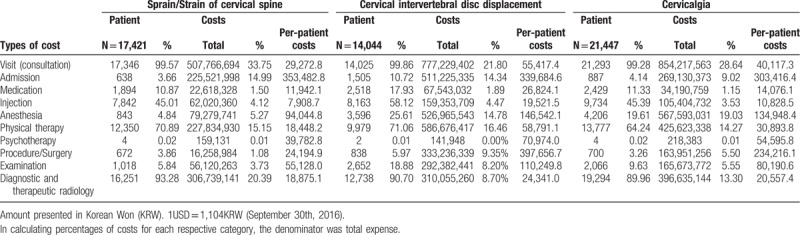
Cost distribution of sprain/strain of cervical spine, cervical intervertebral disc displacement, and cervicalgia.
